# UBA1 knockdown dysregulates UBA1-sensitive proteins and impairs muscle function in models of spinal muscular atrophy X-linked 2

**DOI:** 10.1242/dmm.052935

**Published:** 2026-08-26

**Authors:** Michelle Curley, Flavia A. Graca, Anna Stephan, Wei Wang, Vishwajeeth R. Pagala, Yong-Dong Wang, Anthony A. High, Fabio Demontis

**Affiliations:** ^1^ Department of Developmental Neurobiology, St. Jude Children's Research Hospital, Memphis, TN 38105, USA; ^2^ Center for Proteomics and Metabolomics, St. Jude Children's Research Hospital, Memphis, TN 38105, USA; ^3^ Department of Cellular and Molecular Biology, St. Jude Children's Research Hospital, Memphis, TN 38105, USA

**Keywords:** Skeletal muscle, UBA1, Ubiquitination, Muscle function, SMAX2, Proteostasis

## Abstract

UBA1 is the primary ubiquitin-activating enzyme that initiates ubiquitination, which regulates protein function and turnover. Although UBA1 loss is cell lethal, silent mutations that reduce *UBA1* mRNA levels cause spinal muscular atrophy X-linked 2 (SMAX2), a disorder marked by skeletal muscle weakness and wasting. However, it remains unexplored how UBA1 impacts the muscle proteome, and whether muscle weakness can arise from reducing UBA1 function solely in skeletal muscle. Here, we examined *Drosophila* and mice with muscle-targeted UBA1 knockdown and found that this intervention reduced protein ubiquitination, muscle function and lifespan. Integrated transcriptomic and proteomic analyses indicated that a limited set of proteins is modulated post-transcriptionally by *Uba1* RNA interference (*Uba1*^RNAi^), suggesting that these UBA1-sensitive proteins rely on optimal UBA1 levels for degradation (*Uba1*^RNAi^-upregulated proteins) and stability (*Uba1*^RNAi^-downregulated proteins). Therefore, despite the general function of UBA1 in ubiquitination, UBA1 knockdown alters the levels of relatively few critical proteins, which may contribute to muscle weakness and SMAX2 pathogenesis. Moreover, although SMAX2-linked UBA1 mutations occur ubiquitously, experimental reduction of UBA1 function solely in skeletal muscle recapitulates key disease aspects, highlighting a possible muscle-centric origin of SMAX2.

## Teaser

**Summary:** We established *Drosophila* and mouse models of spinal muscular atrophy X-linked 2 and identified regulated proteins that may contribute to disease pathogenesis.

## INTRODUCTION

Ubiquitination is an important post-translational modification that modulates protein function, localization and degradation (Deol et al., 2019; Hicke, 2001; Komander, 2009; Rape, 2018; Swatek and Komander, 2016). This process involves the coordinated action of a primary E1 ubiquitin-activating enzyme (UBA1, formerly known as UBE1), ∼35 E2 ubiquitin-conjugating enzymes and ∼620 E3 ubiquitin ligases (Collins and Goldberg, 2017; Navon and Ciechanover, 2009; Pohl and Dikic, 2019; Schmidt and Finley, 2014; Schwartz and Ciechanover, 1999). Mono-ubiquitination typically modulates protein import, localization and stability (Deol et al., 2019; Komander, 2009; Livneh et al., 2017; Platta et al., 2007; Rape, 2018; Sadowski et al., 2012; Swatek and Komander, 2016), whereas poly-ubiquitination generally targets proteins for degradation (Deol et al., 2019; Komander, 2009; Nucifora et al., 2016; Ohtake and Tsuchiya, 2017; Ohtake et al., 2018; Swatek and Komander, 2016; Xu et al., 2009b).

UBA1 is expressed ubiquitously across tissues and serves as the principal E1 enzyme that initiates E2-mediated ubiquitin conjugation (Groen and Gillingwater, 2015). Owing to its essential role, complete loss of UBA1 is cell lethal (Kulkarni and Smith, 2008; McGrath et al., 1991). However, a partial decline in UBA1 activity is compatible with cell survival and leads to tissue-specific disease manifestations. For example, silent mutations that decrease *UBA1* mRNA levels are among the hypomorphic mutations that cause spinal muscular atrophy X-linked 2 (SMAX2), a disorder marked by progressive muscle weakness and wasting that lead to respiratory distress and early childhood death (Balak et al., 2017; Dlamini et al., 2013; Jędrzejowska et al., 2015; Öztürk et al., 2022; Ramser et al., 2008; Shaughnessy et al., 2020). Other mutations that decrease UBA1 activity are the cause of VEXAS syndrome, an adult-onset systemic inflammatory condition (Beck et al., 2020; Poulter et al., 2021) that leads to the progressive failure of hematopoietic stem cells (Grayson et al., 2021; Patel et al., 2021; Temple and Kosmider, 2022). Although a partial decline in UBA1 activity is the unifying cause of SMAX2 and VEXAS, it remains largely unexplored how a partial decline in UBA1 activity and ubiquitination affects the proteome and disease progression. Because ubiquitination regulates a vast number of (virtually all) proteins, UBA1 reduction may globally disrupt protein ubiquitination and lead to pervasive proteomic changes. Alternatively, it may impact subsets of proteins that are particularly sensitive to even relatively minor perturbations in UBA1 function.

We previously examined the outcome of moderate *UBA1* RNA interference (RNAi) (∼20% knockdown) in human cultured HEK293T cells and found that a limited set of proteins is modulated, including peroxisomal proteins and other organelle components (Hunt et al., 2023), suggesting that a subset of UBA1-sensitive proteins is primarily affected by UBA1 knockdown. However, these studies were conducted in cultured cells; therefore, it remains undetermined whether selective proteomic changes in limited protein subsets also occur *in vivo*. In the case of SMAX2, it is also unclear whether defective UBA1 levels in skeletal muscle are a cause of muscle weakness or, alternatively, whether this arises from defective UBA1 activity in other tissues, such as motor neurons or brain areas that control movement.

To address these questions, we utilized the fruit fly *Drosophila melanogaster* and mice to examine the impact of UBA1 knockdown in skeletal muscle, which is relevant for SMAX2 because this disease is characterized by muscle weakness (Balak et al., 2017; Dlamini et al., 2013; Jędrzejowska et al., 2015; Öztürk et al., 2022; Ramser et al., 2008; Shaughnessy et al., 2020) and can arise from C1731T (Asn577Asn) silent/synonymous mutations that do not change the UBA1 protein sequence but decrease *UBA1* mRNA levels (Balak et al., 2017; Dlamini et al., 2013; Jędrzejowska et al., 2015; Öztürk et al., 2022; Ramser et al., 2008; Shaughnessy et al., 2020), presumably because of decreased enhancer recruitment (Plank and Dean, 2014). We found that muscle-restricted UBA1 knockdown reduces muscle strength in both organisms. Mechanistically, this occurs by reducing overall protein ubiquitination, but this affects the levels of only limited sets of proteins. Altogether, these analyses identify key proteins that are modulated post-transcriptionally by UBA1 knockdown and indicate that decline in UBA1 levels restricted to skeletal muscle causes muscle weakness by impacting UBA1-sensitive proteins.

## RESULTS

### Muscle-specific knockdown of Uba1 in skeletal muscle reduces protein ubiquitination, muscle function and lifespan in *Drosophila*

The fruit fly *D. melanogaster* is utilized to model many human diseases (Bellen et al., 2019), and it was previously employed to examine the role of components of the ubiquitin–proteasome system in skeletal muscle (Hunt et al., 2025a, 2021b) and myopathies (Cobb et al., 2023; Damschroder et al., 2022; Poudel et al., 2025; Richardson et al., 2024; Sujkowski et al., 2022; Sujkowski and Wessells, 2018).

**Fig. 1.**
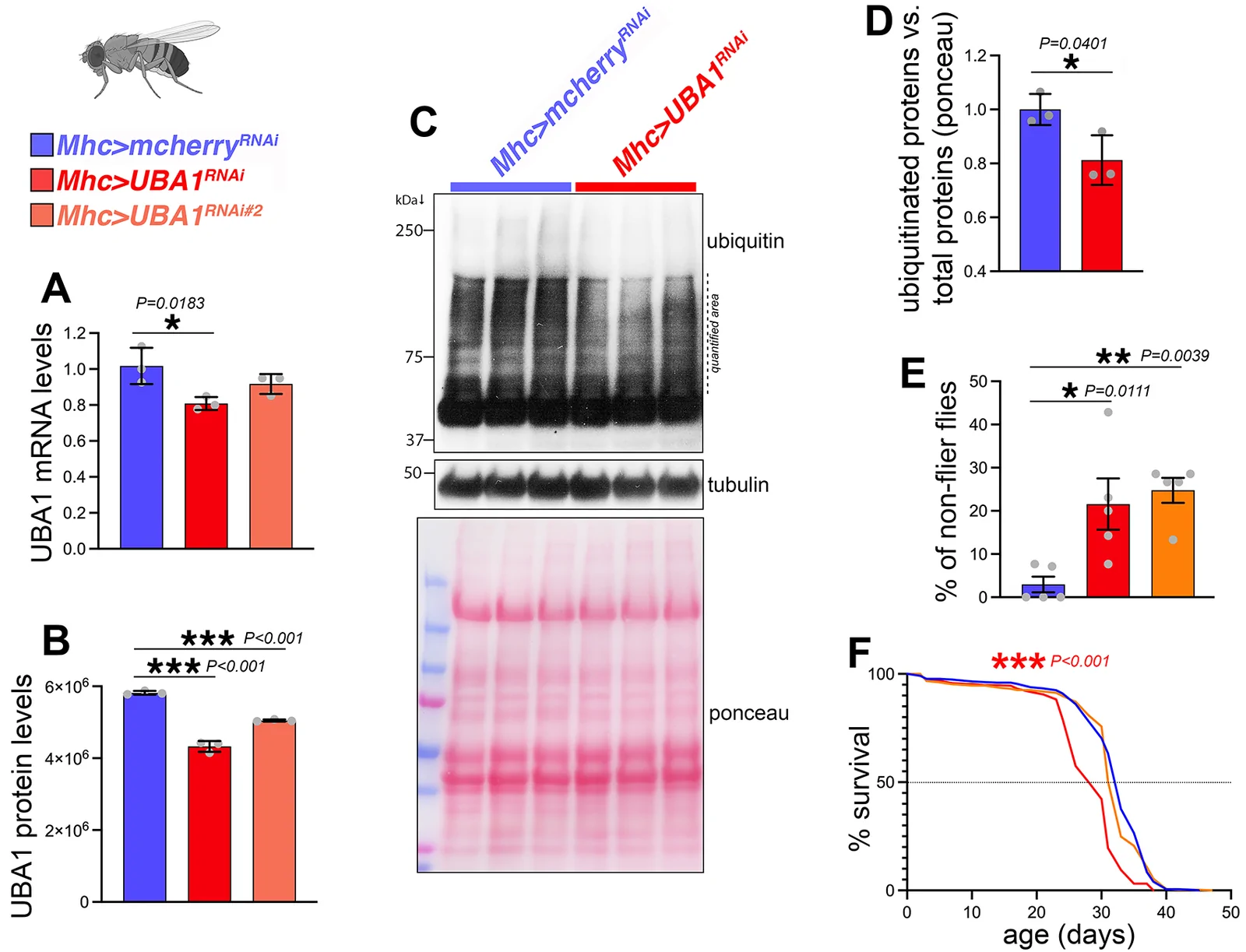
**Uba1 knockdown in *Drosophila* skeletal muscle reduces protein ubiquitination, flight capacity and lifespan.** The skeletal muscle-specific Mhc-Gal4 driver was utilized to drive the expression of two distinct *Uba1* RNA interference (RNAi) lines in the skeletal muscle of adult flies. (A,B) Compared to control *mcherry*^RNAi^, Uba1 knockdown leads to a decline in *Uba1* mRNA (A; fold change) and protein (B; reporter ion intensities) levels, as estimated by quantitative reverse transcription PCR (qRT-PCR) and mass spectrometry, respectively. *Uba1*^RNAi (BL#76066)^ triggers stronger Uba1 knockdown than *Uba1*^RNAi#2 (BL#25957)^. The graphs display the mean±s.d. and *n*=3 biological replicates (a pool of >10 thoraces/replicate). *P*-values were calculated with one-way ANOVA with Dunnett's multiple comparisons test, *P<0.05 and ***P<0.001. Fly scheme was created in BioRender by Demontis, F. (2026). https://BioRender.com/1i8vuo7. This figure was sublicensed under CC-BY 4.0 terms. (C,D) Western blot of thoracic skeletal muscles from flies with muscle-specific *Uba1*^RNAi^ and control *mcherry*^RNAi^. Probing with anti-ubiquitin antibodies (C) indicates that Uba1 knockdown significantly reduces the overall levels of poly-ubiquitinated proteins (D), as indicated by the normalization with total protein levels (detected by Ponceau S staining). The graphs display the mean±s.d. and *N*=3 biological replicates (a pool of >10 thoraces/replicate). *P*-values were calculated with an unpaired two-tailed *t*-test, *P<0.05. (E) Analysis of skeletal muscle function with flight assays indicates that Uba1 knockdown reduces muscle function compared to control *mcherry*^RNAi^, as indicated by the number of 10-day-old flies unable to fly. The graphs display the mean±s.e.m. and *N*=5 (each consisting of a group of 12-15 flies). *P*-values were calculated with one-way ANOVA with Dunnett's multiple comparisons test, *P<0.05 and **P<0.01. (F) Survival analysis at 29°C indicates that muscle-specific Uba1 knockdown obtained with the *Uba1*^RNAi (BL#76066)^ line (*n*=348 flies) significantly (***P<0.001; log-rank test) shortens lifespan (median lifespan, ∼28 days) compared to control *mcherry*^RNAi^ (*n*=343 flies; median lifespan, ∼32 days), whereas a minor non-significant reduction is observed with the weaker *Uba1*^RNAi#2 (BL#25957)^ line (*n*=334 flies; median lifespan, ∼31 days).

Previously, we found that Uba1 knockdown in developing larval body wall muscles by using Mef2-Gal4 reduces muscle size and causes developmental lethality (Hunt et al., 2019b). To avoid similar developmental lethality, here we utilized a Mhc-Gal4 line that drives minimal or no transgene expression during development (Demontis et al., 2014; Demontis and Perrimon, 2010; Rai et al., 2021; Schuster et al., 1996). On this basis, this muscle-specific Mhc-Gal4 driver was used to induce the transgenic expression of two distinct RNAi lines that target Uba1 and to determine the consequent outcome on skeletal muscle function, compared to control *mcherry*^RNAi^. SMAX2 is predominantly seen in male infants because it is an X-linked recessive disorder (Balak et al., 2017; Dlamini et al., 2013; Jędrzejowska et al., 2015; Öztürk et al., 2022; Ramser et al., 2008; Shaughnessy et al., 2020). Therefore, Uba1 knockdown was induced in the skeletal muscle of male flies, which were then analyzed at the age of 10 days, i.e. early in the fly's lifespan (akin to the stage at which SMAX2 manifests in humans). These analyses indicated that, depending on the RNA line, UBA1 knockdown reduces *Uba1* mRNA levels by 20% and 9% in the skeletal muscle of 10-day-old flies (Fig. 1A), and this results in a significant decline in Uba1 protein levels by 26% and 13%, respectively (Fig. 1B), as assessed by quantitative reverse transcription PCR (qRT-PCR) and mass spectrometry (MS).

Considering that UBA1 is the main E1 ubiquitin-activating enzyme (Groen and Gillingwater, 2015), we next examined the impact of Uba1 knockdown on protein ubiquitination. Western blot analyses with anti-ubiquitin antibodies indicated that *Uba1*^RNAi^ significantly reduced (−19%) overall protein ubiquitination (Fig. 1C,D). On this basis, having established a model with muscle-restricted moderate knockdown of Uba1, we next examined the outcome for muscle function. The bulk of *Drosophila* muscle is normally utilized for flying (Demontis et al., 2013b), and analysis of the flight capacity indicated a significantly higher percentage of flies unable to fly when Uba1 levels were reduced specifically in skeletal muscle, compared to control flies with *mcherry*^RNAi^ (Fig. 1E).

Previous studies indicate that a decline in muscle proteostasis and strength can impair organismal fitness and reduce lifespan (Jiao et al., 2023; Jiao and Demontis, 2017), in agreement with the epidemiological observations that muscle weakness increases the risk of mortality from all causes in humans (Metter et al., 2002; Ruiz et al., 2008). In line with these findings, Uba1 knockdown induced a significant decline in the median lifespan (i.e. the day at which 50% of flies were alive), although this occurred when driving the strongest transgenic RNAi line (*Uba1*^RNAi^) but not with the weaker *Uba1*^RNAi#2^ (Fig. 1F). Altogether, these findings indicated that muscle-targeted, partial decline in Uba1 levels reduces overall protein ubiquitination, muscle function and lifespan.

### Uba1 knockdown impacts the levels of a limited set of Uba1-sensitive proteins in *Drosophila* skeletal muscles

**Fig. 2.**
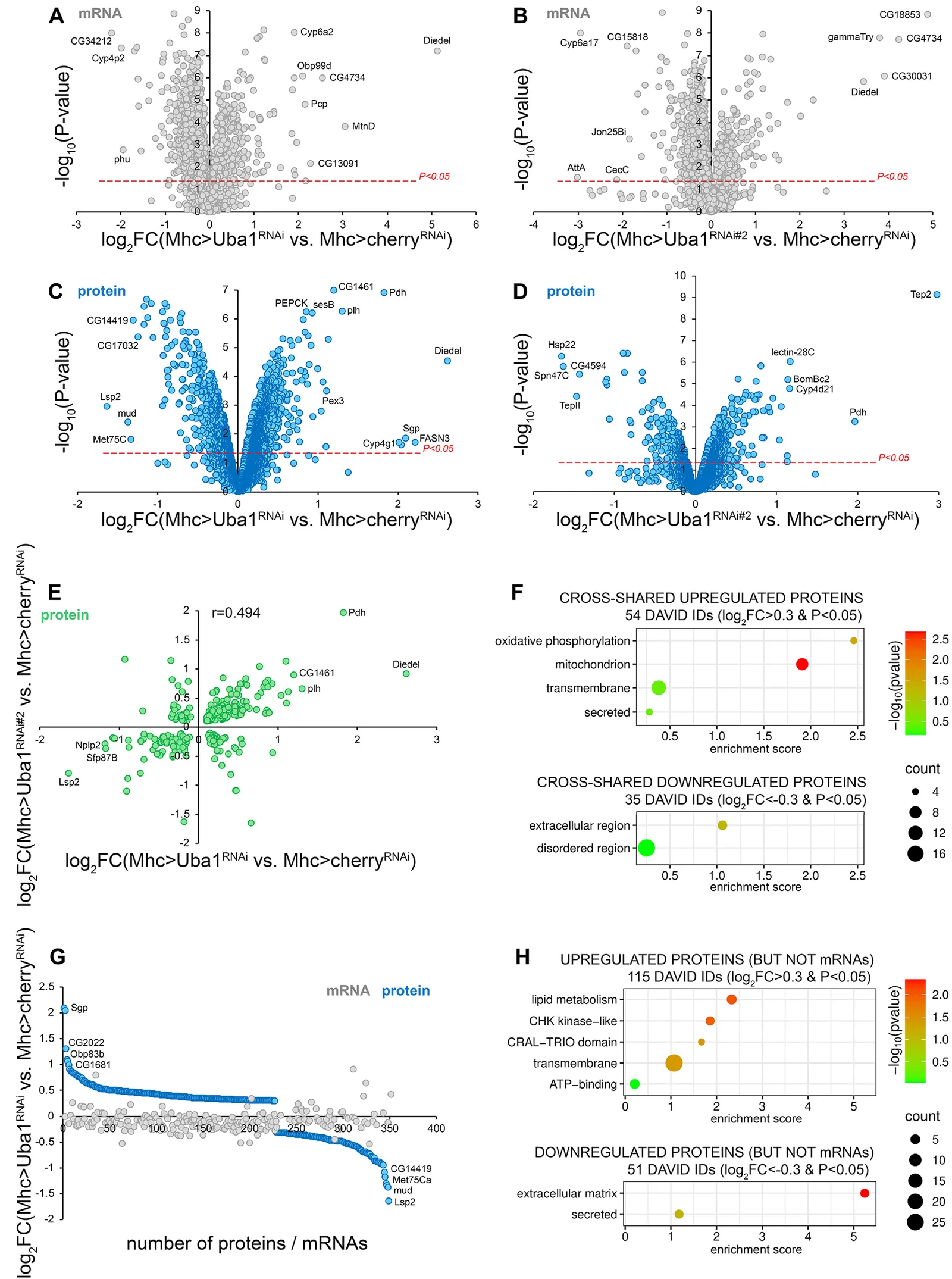
**Uba1 knockdown alters the abundance of a proteome subset consisting of Uba1-sensitive proteins in *Drosophila* skeletal muscle.** (A,B) RNA-sequencing (RNA-seq) analysis of the transcriptional changes induced by *Uba1*^RNAi^ (A) and *Uba1*^RNAi#2^ (B) in *Drosophila* skeletal muscles (*n*=10,202 mRNAs). The *x*-axis reports the log_2_FC(*Uba1*^RNAi^ versus *mcherry*^RNAi^), whereas the *y*-axis reports the −log_10_(*P*-value), which were examined with *N*=3 biological replicates (each consisting of >10 thoraces) per group. (C,D) Tandem mass tag (TMT) mass spectrometry identifies the protein changes induced with muscle-specific Uba1 knockdown, obtained with the *Uba1*^RNAi^ (C) and *Uba1*^RNAi#2^ (D) lines, compared to control *mcherry*^RNAi^. Despite a global decline in protein ubiquitination (Fig. 1C,D), *Uba1* RNAi significantly alters the levels of relatively few proteins (here defined as Uba1-sensitive proteins). The *x*-axis reports the log_2_FC(*Uba1*^RNAi^ versus *mcherry*^RNAi^), whereas the *y*-axis reports the −log_10_(*P*-value); *N*=3/group; *n*=5465 proteins were quantified by these TMT analyses. (E) Cross-comparison of the protein changes that are significantly induced (*P*<0.05; no log_2_FC cut-off) by both RNAi lines that trigger Uba1 knockdown (*n*=356 proteins). There is an overall good correlation in the proteomic changes induced by *Uba1*^RNAi^ and *Uba1*^RNAi#2^ versus control *mcherry*^RNAi^ (*r*=0.494). The *x*-axis reports the log_2_FC(*Uba1*^RNAi^ versus *mcherry*^RNAi^), whereas the *y*-axis reports the log_2_FC(*Uba1*^RNAi#2^ versus *mcherry*^RNAi^). (F) Analysis of the Gene Ontology (GO) categories that are enriched in the cross-shared proteins that are significantly upregulated (log_2_FC>0.3 and *P*<0.05) and downregulated (log_2_FC<−0.3 and *P*<0.05) by both *Uba1* RNAi lines. The enrichment score, count and −log_10_(*P*-value) are indicated. (G) Analysis of the proteins that are significantly modulated (0.3<log_2_FC<−0.3 and *P*<0.05) at the protein, but not mRNA, level by Uba1 knockdown (*n*=349). The *y*-axis reports the log_2_FC(*Uba1*^RNAi^ versus *mcherry*^RNAi^) for proteins (blue) and corresponding mRNAs (gray), whereas the *x*-axis reports the proteins in descending order of regulation. (H) Analysis of the GO categories that are enriched in the proteins that are significantly upregulated (log_2_FC>0.3 and *P*<0.05) and downregulated (log_2_FC<−0.3 and *P*<0.05) at the protein, but not mRNA, level by *Uba1*^RNAi^. The enrichment score, count and −log_10_(*P*-value) are indicated. A total of *n*=166 proteins (DAVID IDs) was analyzed.

To determine the mechanisms underlying the decline in muscle function in response to Uba1 knockdown, we next utilized RNA sequencing (RNA-seq) (Dataset 1) and tandem mass tag (TMT) MS (Dataset 2) to determine the changes in mRNA and protein levels that are induced by *Uba1*^RNAi^ versus control *mcherry*^RNAi^ in skeletal muscle. There were several gene expression changes induced by Uba1 knockdown that may represent stress responses that sustain muscle homeostasis (Fig. 2A,B) and that partially overlap with changes in protein levels (Fig. 2C,D). For example, immune regulators and secreted proteins were among the genes concordantly upregulated by both *Uba1* RNAi lines (Fig. S1A, Fig. S2A, Dataset 3). Comparison of the protein changes induced by *Uba1*^RNAi^ and *Uba1*^RNAi#2^ versus control *mcherry*^RNAi^ indicated the induction of overlapping responses (Fig. 2E), although the correlation (*r*=0.494) between *Uba1*^RNAi^ and *Uba1*^RNAi#2^ is likely to be limited by the different potency of the two distinct RNAi lines (Fig. 1A,B). Common *Uba1*^RNAi^-upregulated proteins included regulators of oxidative phosphorylation, mitochondrial, transmembrane and secreted proteins, whereas proteins downregulated by *Uba1*^RNAi^ included extracellular and disordered proteins (Fig. 2F).

Although modulation of some of these proteins may arise from transcriptional changes (Fig. 2A,B), others were modulated at the protein, but not mRNA, level, and this post-transcriptional modulation could result from their decreased ubiquitination. In particular, proteins that are upregulated in response to Uba1 knockdown may strictly rely on optimal Uba1 levels for ubiquitination and turnover and, therefore, may accumulate because of decreased poly-ubiquitin-driven degradation. However, ubiquitination can also ensure proper localization and stability of modified proteins (Hunt et al., 2025b; Rodrigues et al., 2013; Srivastava and Chakrabarti, 2014; Whitcomb et al., 2019); therefore, Uba1 knockdown (and the consequent decline in regulatory ubiquitination) may lead to protein instability and downregulation.

Consistent with this scenario, the proteins modulated by *Uba1*^RNAi^ post-transcriptionally (i.e. without corresponding changes in mRNA levels) included 227 upregulated and 122 downregulated proteins (Fig. 2G). Upregulated proteins included the peroxin Pex3, homologous to PEX3 (Farre et al., 2019), and the peroxisomal enzyme Sgp, homologous to the fatty acyl-CoA reductases FAR1/2, which catalyze the reduction of fatty acids to fatty alcohols, a process required for the synthesis of ether lipids (Honsho and Fujiki, 2017). More broadly, Gene Ontology (GO) categories enriched among *Uba1*^RNAi^-upregulated proteins included lipid metabolism and transmembrane proteins (Fig. 2H). Downregulated proteins were enriched for secreted and extracellular factors, such as Lsp2, but also included intracellular proteins such as Mud, homologous to human golgin A3 (Lowe, 2019) (Fig. 2G,H). Altogether, these findings indicated that moderate knockdown of Uba1 in *Drosophila* skeletal muscles results in upregulation and downregulation of a limited subset of Uba1-sensitive proteins, which may require optimal levels and activity of Uba1 for their turnover (*Uba1*^RNAi^-upregulated proteins) or stability (*Uba1*^RNAi^-downregulated proteins). However, in addition to modulation via Uba1-initiated ubiquitination, it is also possible that some of these proteins are modulated indirectly, i.e. via mechanisms other than ubiquitination, such as changes in protein translation.

### UBA1 knockdown reduces protein ubiquitination, muscle mass and force production in mice

**Fig. 3.**
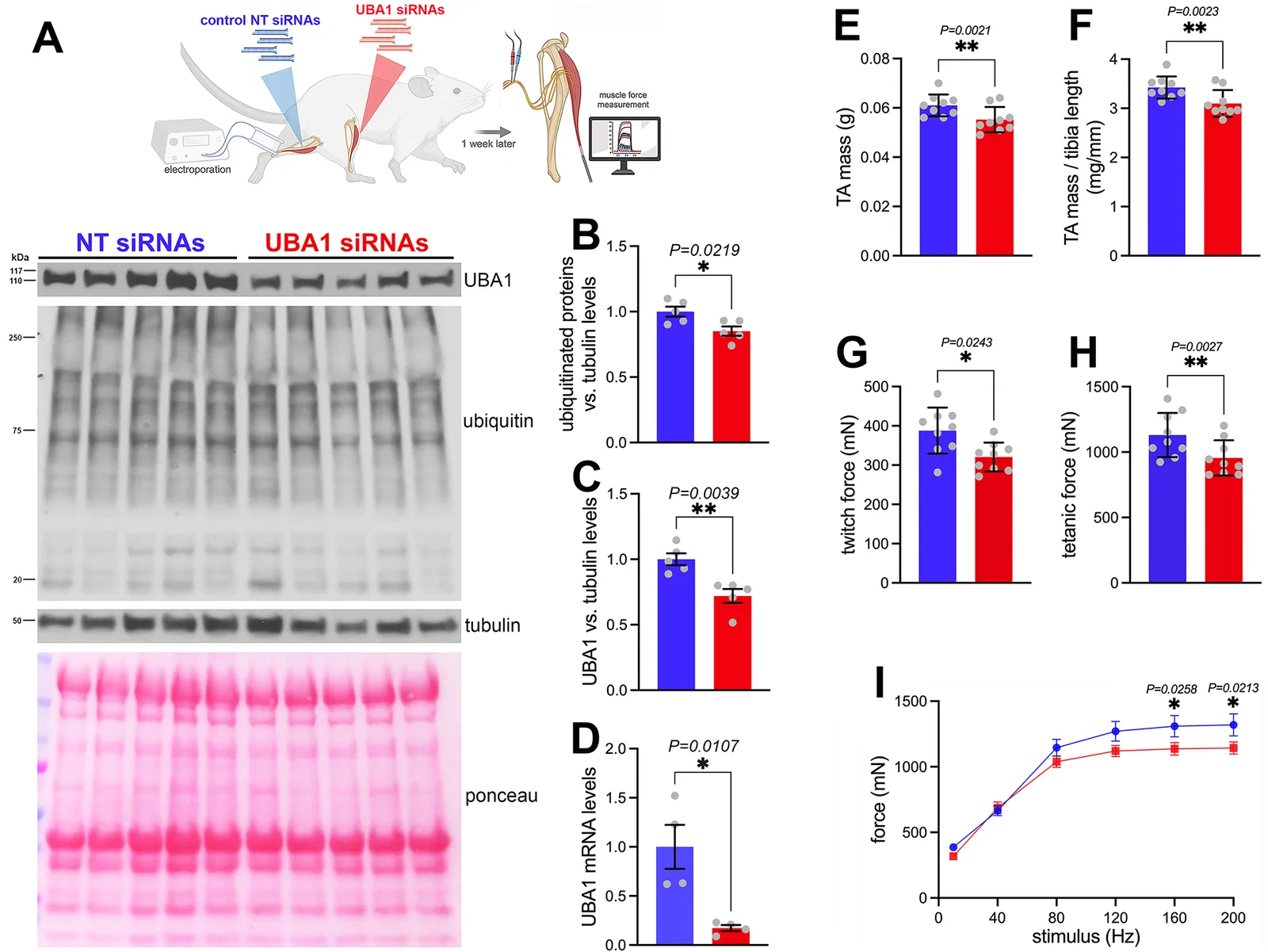
**UBA1 knockdown in mouse skeletal muscle reduces protein ubiquitination, muscle mass and muscle force production.** (A) Electroporation of control non-targeting (NT) siRNAs (blue) and *Uba1* siRNAs (red). Contralateral tibialis anterior (TA) muscles of 3-month-old male mice were electroporated with a mixture of four distinct siRNA species (SMARTpool) for the same target. The scheme was created in BioRender by Demontis, F. (2026). https://BioRender.com/4x08m3h. This figure was sublicensed under CC-BY 4.0 terms. (B,C) Western blot of contralateral mouse TA muscles electroporated with control NT siRNAs and *Uba1* siRNAs. UBA1 protein levels and UBA1 function (i.e. protein ubiquitination) are significantly reduced by UBA1 knockdown versus controls (C). The graph displays the mean±s.e.m. with *n*=5 muscles/group sourced from *N*=5 mice; *P<0.05 (unpaired two-tailed *t*-test). (D) qRT-PCR indicates that *Uba1* siRNAs (red) reduce *Uba1* mRNA levels compared to control NT siRNAs (blue). The graph displays the mean±s.e.m. with *N*=4 biological replicates; *P<0.05 (unpaired two-tailed Student's *t*-test). (E,F) *Uba1* siRNAs (red) reduce the TA muscle weight (E) compared to NT siRNAs (blue), also when normalized by the length of the tibia bone (F). (G-I) UBA1 knockdown reduces muscle force production compared to NT siRNAs, as indicated by the measurement of the twitch force (G), the tetanic force (H) and the force frequency (I). The graphs display the mean±s.e.m. with *n*=9 muscles/group sourced from *N*=9 mice; *P<0.05, **P<0.01, calculated with the paired two-tailed Student's *t*-test (E-H) and two-way ANOVA with Tukey's multiple comparisons test (I).

Having established the importance of Uba1 for muscle function in *Drosophila*, we next examined whether it plays a similar role in mouse skeletal muscles. To this purpose, we utilized electroporation of siRNA SMARTpools into the tibialis anterior (TA) muscles (Donà et al., 2003; Hunt et al., 2021b; Sandri et al., 2003, 2004; Stephan et al., 2022) of 3-month-old C57BL/6J male mice. This age roughly corresponds to early childhood, and it is a stage at which postnatal muscle growth is still occurring (White et al., 2010). Specifically, *Uba1* siRNAs were electroporated into one TA muscle, whereas the contralateral TA muscle was electroporated with control non-targeting (NT) siRNAs (Fig. 3A). A week after electroporation, the TA muscles were dissected and examined by western blotting. As expected, UBA1 knockdown reduced UBA1 protein (Fig. 3B,C) and mRNA (Fig. 3D) levels compared to control NT siRNAs. Moreover, there was also a significant decline in the overall levels of protein ubiquitination following UBA1 knockdown (Fig. 3B,C), as found in *Drosophila* (Fig. 1C,D). Altogether, these findings indicate that siRNA electroporation is effective at reducing UBA1 levels and activity, as indicated by the decline in protein ubiquitination.

In this model, we next examined the functional outcome of partial UBA1 knockdown in skeletal muscle. Analysis of TA muscles indicated that UBA1 knockdown induced a significant decline in TA weight compared to that of controls. Furthermore, this decrease in muscle mass remained significant when normalized by the length of the associated tibia bone (Fig. 3E,F). *In situ* muscle force measurements (Graca et al., 2023a; Hunt et al., 2021b, 2019b; Liu et al., 2016) indicated that UBA1 knockdown reduced the twitch force (i.e. the force generated with a physiological stimulation; Fig. 3G), the tetanic force (i.e. the force generated with a maximal stimulation; Fig. 3H) and the isometric force frequency (i.e. the force produced when increasing the frequency of electrical stimulation while maintaining constant the muscle length; Fig. 3I). Altogether, these findings indicate that a partial decline in UBA1 levels reduces muscle mass and impairs muscle force production.

**Fig. 4.**
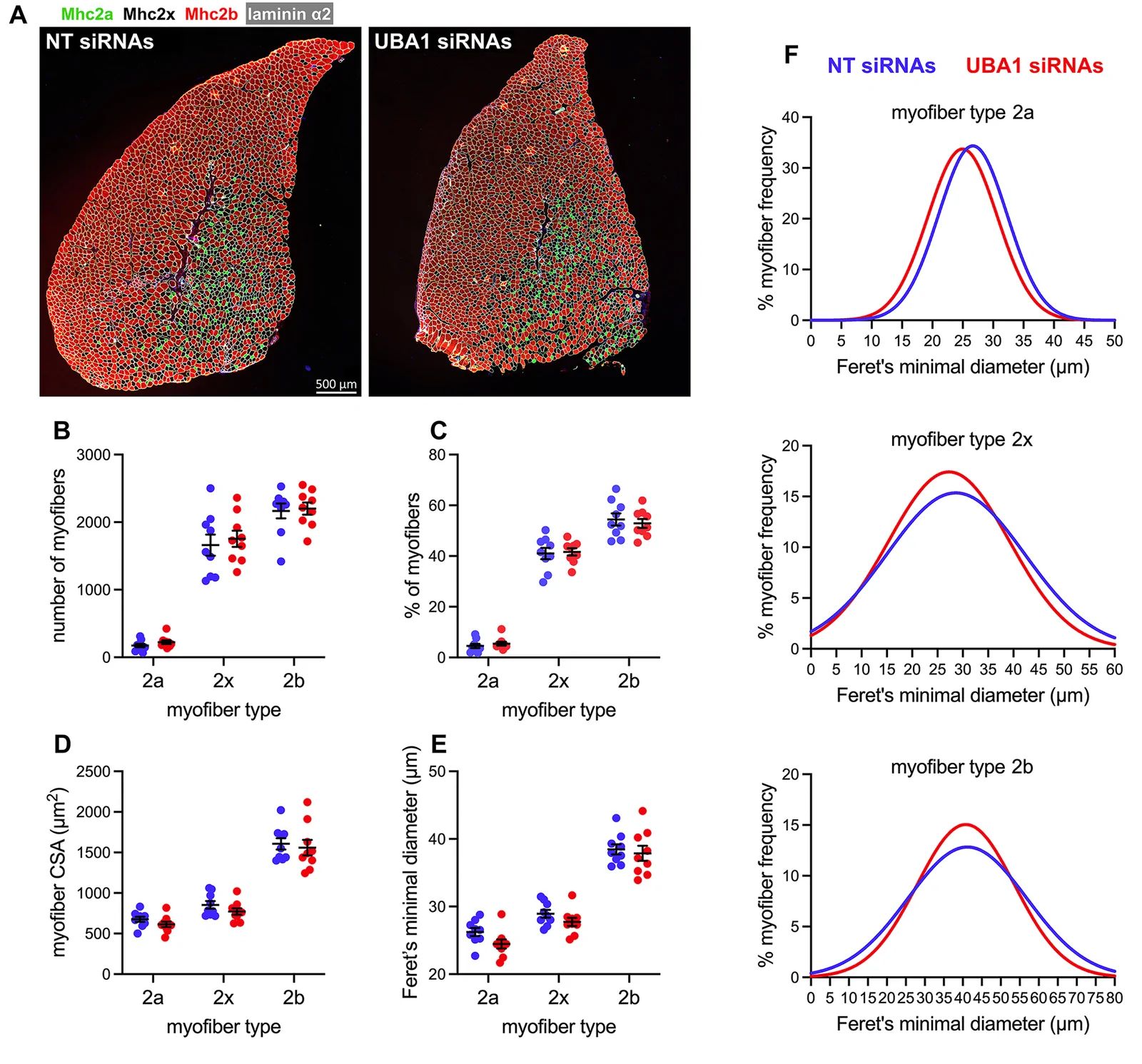
**UBA1 knockdown marginally reduces myofiber size in the TA skeletal muscle.** (A) Immunostaining of transverse sections from contralateral TA skeletal muscles electroporated with control NT siRNAs and *Uba1* siRNAs. Myosin heavy chain isoforms are immunodetected to distinguish type 2a (MHC2a-positive; green), type 2b (MHC2b-positive; red) and type 2x (MHC2a/2b-negative; black) myofibers. Myofiber boundaries are identified by immunoreactivity for laminin α2 (white). (B,C) The number of myofibers (B) and the proportion of distinct myofiber types (C) are not affected by *Uba1* siRNAs (red) versus control NT siRNAs (blue). (D-F) Analysis of the myofiber cross-sectional area (CSA; D) and Feret's minimal diameters (E) for type 2a, type 2x and type 2b myofibers indicates that there is a non-significant trend towards lower myofiber sizes in response to *Uba1* siRNAs (red) versus NT siRNAs (blue), as also shown by the Gaussian distributions of myofiber sizes (F). The graphs display the mean±s.e.m. from *N*=9 biological replicates (muscles) per group, obtained from *n*=9 independent mice. Statistical analysis was done with two-way ANOVA with Sidak's multiple comparison test.

Shifts in skeletal muscle mass that occur postnatally typically arise from corresponding changes in the size of myofibers, but can also be caused by a variation in the number of myofibers or in the prevalence of different myofiber types that have distinct sizes (Demontis et al., 2013b; Gilmore et al., 2023; Piccirillo et al., 2014). On this basis, transverse sections of TA muscles that were electroporated with *Uba1* siRNAs versus NT siRNAs were immunostained (Fig. 4A) to determine whether UBA1 knockdown induces any changes in myofiber size, type and number. Antibodies specific for different myosin heavy chain (MHC) isoforms were utilized to detect distinct myofiber types that are present in TA muscles: type 2a, type 2x and type 2b myofibers (Hunt et al., 2021a,b, 2019b). Analysis of the number (Fig. 4B) and type (Fig. 4C) of myofibers revealed no substantial changes in muscles electroporated with *Uba1* siRNAs versus NT siRNAs. There was a trend towards lower sizes (albeit not significant) for all myofiber types in response to UBA1 knockdown, as estimated from the analysis of the cross-sectional area (Fig. 4D) and the Feret's minimal diameter (Fig. 4E), and also indicated by the Gaussian distributions (Fig. 4F), suggesting that decreased myofiber size may marginally contribute to the decline in muscle mass observed in response to UBA1 knockdown.

### UBA1 knockdown affects a subset of the skeletal muscle proteome in mice

Reducing UBA1 levels and activity may compromise muscle function because of the generalized decline in protein ubiquitination and the consequent impact on the levels and function of a myriad of UBA1 target proteins. Alternatively, as observed in *Drosophila* (Fig. 2), many proteins may be resilient to UBA1 knockdown, and the muscle force impairment may instead result from circumscribed changes in relatively few proteins.

**Fig. 5.**
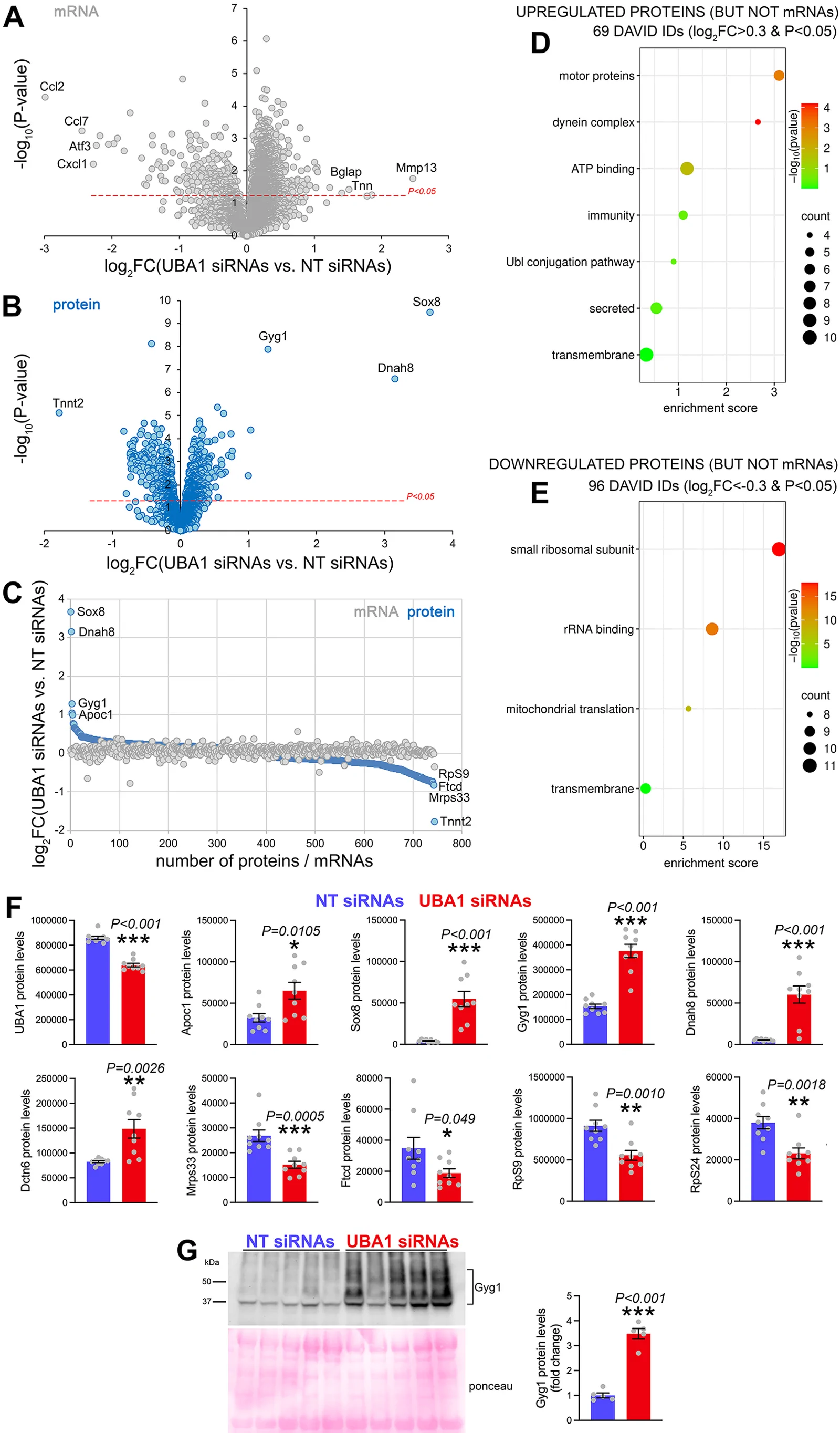
**Electroporation of *Uba1* siRNAs alters the levels of a subset of UBA1-sensitive proteins in mouse skeletal muscle.** (A,B) RNA-seq (A; *n*=12,326 mRNAs) and TMT mass spectrometry (B; *n*=4077 proteins) analyses of the changes induced by the electroporation of *Uba1* siRNAs versus control NT (non-targeting) siRNAs in contralateral TA skeletal muscles. The *x*-axis reports the log_2_FC(*Uba1* siRNAs versus NT siRNAs), whereas the *y*-axis reports the −log_10_(*P*-value), which were examined with *N*=4 (RNA-seq) and *N*=9 (TMT) biological replicates (i.e. muscles) per group. (C) Protein changes (blue; *n*=744 proteins) that are induced (*P*<0.05; no log_2_FC cut-off) by *Uba1* siRNAs versus control NT siRNAs without any significant and/or corresponding changes in mRNA levels (gray). The number of proteins and mRNAs is listed on the *x*-axis in descending order, whereas the log_2_FC is shown on the *y*-axis. (D,E) Gene Ontology (GO) categories enriched among the proteins that are significantly upregulated (log_2_FC>0.3 and *P*<0.05) and downregulated (log_2_FC<−0.3 and *P*<0.05) by *Uba1* siRNAs versus control NT siRNAs but that are not correspondingly modulated at the mRNA level. The enrichment score, count and −log_10_(*P*-value) are indicated. A total of *n*=165 proteins (DAVID IDs) was analyzed. (F) Examples of proteins upregulated by UBA1 knockdown independently from changes in mRNA levels include Apoc1, Sox8, Gyg1, Dnah8 and Dctn6: these proteins may require optimal UBA1 levels for their poly-ubiquitination and turnover and, hence, accumulate in response to UBA1 knockdown. Conversely, the levels of Mrps33, Ftcd, Rps9 and Rps24 decrease in response to *Uba1* RNAi, suggesting that these proteins strictly depend on UBA1 for their stability. The graphs display the mean±s.e.m. with *n*=9 muscles/group sourced from *N*=9 mice; *P<0.05, **P<0.01, ***P<0.001 (unpaired two-tailed *t*-test). (G) Western blot of contralateral TA muscles electroporated with control NT siRNAs and *Uba1* siRNAs (same samples analyzed in Fig. 3A-D). The protein levels of glycogenin-1 (Gyg1) normalized by Ponceau S levels significantly increase in response to UBA1 knockdown. The graph displays the mean±s.e.m. with *n*=5 muscles/group sourced from *N*=5 mice; ***P<0.001 (unpaired two-tailed Student's *t*-test).

To test these hypotheses, we utilized RNA-seq (Dataset 4) and TMT MS (Dataset 5) to examine the transcriptional and proteomic changes induced by UBA1 knockdown in mouse skeletal muscles. There were several gene expression changes induced by *Uba1* siRNAs versus control NT siRNAs (Fig. 5A) that may arise from an adaptive response to decreased ubiquitination (Rai et al., 2025b). Significant mRNA changes included the upregulation of extracellular matrix components and the downregulation of transcription factors and chromatin remodelers (Fig. S1B). However, there was a minimal correlation between this significant set of gene expression changes and the alteration of the levels of the corresponding proteins (Fig. S2B, Dataset 3), presumably because of balancing changes in mRNA translation and protein degradation that can disconnect the transcriptome from the proteome (Ghazalpour et al., 2011; Gygi et al., 1999; Hunt et al., 2019a; Yeung, 2011). Moreover, the mRNAs that were significantly regulated by Uba1 knockdown in *Drosophila* muscle were not homologous to those modulated in mice (Dataset 3).

Despite an overall decline in protein ubiquitination caused by *Uba1* RNAi (Fig. 3B,C), there were limited changes at the proteome level (Fig. 5B), and only a restricted subset of *Uba1*^RNAi^-regulated proteins was modulated post-transcriptionally without underlying corresponding mRNA changes (Fig. 5C). These proteins included both upregulated and downregulated proteins (Fig. 5D,E), which were, respectively, enriched for motor proteins of the dynein complex (e.g. Dnah8 and Dctn6) and regulators of translation (e.g. Rps9 and Rps24) (Fig. 5D-F). Additional examples of upregulated proteins include the apolipoprotein Apoc1, the transcription factor Sox8 and the glycogenin Gyg1 (a glycosyltransferase that forms a short glucose polymer that acts as primer to initiate glycogen biosynthesis), whereas downregulated proteins also include the mitochondrial ribosomal protein S33 (Mrps33) and the formimidoyltransferase cyclodeaminase enzyme (Ftcd), involved in histidine degradation. Western blots with antibodies for the glycogenin Gyg1 further confirmed that the levels of this protein increase in muscles with UBA1 knockdown versus contralateral controls (Fig. 5G). Altogether, these analyses indicate that a moderate decline in UBA1 levels impacts the abundance of a limited subset of muscle proteins, despite an overall decline in ubiquitination.

### Largely different UBA1-sensitive proteins are modulated by UBA1 knockdown in *Drosophila* versus mouse skeletal muscles

**Fig. 6.**
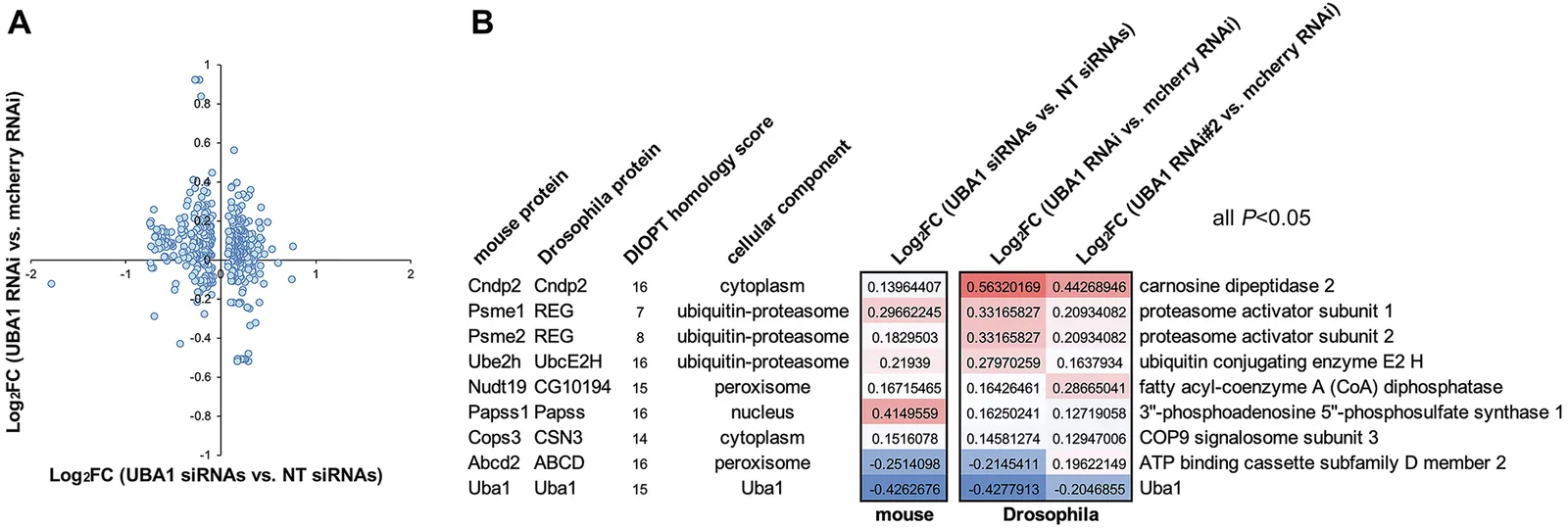
**Comparison of the mouse and *Drosophila* UBA1-sensitive proteomes.** (A) Analysis of the proteins that are significantly (*P*<0.05) modulated by *Uba1* siRNAs versus NT siRNAs in mouse skeletal muscles (*x*-axis; log_2_FC values), and the corresponding regulation of the *Drosophila* homologs in the muscles with *Uba1* RNAi versus control *mcherry* RNAi (*y*-axis; log_2_FC values, no *P*-value cut-off). (B) Apart from UBA1, eight proteins are consistently and significantly modulated by UBA1 knockdown in mouse and *Drosophila* skeletal muscles: these include components of the ubiquitin–proteasome system and of peroxisomes. The mouse and *Drosophila* homolog protein names are listed alongside the homology score obtained with DIOPT, the cellular location/component and the fold changes (log_2_FC; all significant, *P*<0.05).

We next examined the UBA1-sensitive proteins that are modulated in *Drosophila* versus mouse skeletal muscle in response to UBA1 knockdown. To this purpose, we retrieved mouse homologs of *Drosophila* Uba1-modulated proteins via the DRSC Integrative Ortholog Prediction Tool (DIOPT) and aligned the changes induced by UBA1 knockdown in mouse versus *Drosophila*. This analysis revealed no consistent pattern, suggesting that Uba1 knockdown regulates largely distinct subsets of proteins in *Drosophila* flight muscles compared to mouse TA muscles (Fig. 6A). However, there were eight proteins that were significantly and consistently regulated by UBA1 knockdown in *Drosophila* and mice. These included primarily upregulated proteins, suggesting that they strictly rely on optimal UBA1 levels for their turnover. These included components of the 11S regulator complex (PA28) of the proteasome (Psme1/Psme2/REG), the ubiquitin conjugating enzyme UBE2H and the peroxisomal enzyme Nudt19/CG10194 (fatty acyl-coenzyme A diphosphatase). However, the peroxisomal protein Abcd2/ABCD (ATP binding cassette subfamily D member 2) was consistently downregulated, indicating that UBA1 is necessary for its stability in *Drosophila*, as also found in mouse muscles. Although these proteins were consistently modulated in *Drosophila* versus mice (Fig. 6), they are not the most strikingly regulated proteins in either organism (Figs 2 and 5). Therefore, although UBA1 knockdown leads to the same phenotypic manifestation (i.e. muscle weakness) in both mouse and *Drosophila*, this occurs via the modulation of largely distinct UBA1-sensitive proteins in each organism. Moreover, in both *Drosophila* and mice, only a minority of the muscle proteome is impacted by a partial UBA1 knockdown.

## DISCUSSION

In this study, we examined the impact of moderate UBA1 decline in mouse and *Drosophila* skeletal muscle and found that, despite a significant reduction in overall protein ubiquitination, the levels of relatively few proteins are modulated by UBA1 knockdown. UBA1 is a key regulator of protein ubiquitination because of its capacity to activate ubiquitin and initiate this process (Groen and Gillingwater, 2015). It was previously estimated that ∼43% of all proteins are normally ubiquitinated (Hansen et al., 2021) and that >99% of proteins carry lysine residues that are potentially available for this modification (Swatek and Komander, 2016). Therefore, even minor disruptions in the function of the ubiquitin-activating enzyme UBA1 may have a pervasive impact on the ubiquitination and levels of many proteins. However, we find that a moderate decline in UBA1 levels decreases protein ubiquitination and reduces muscle strength (Figs 1 and 3) but affects the abundance of a limited set of proteins (Figs 2 and 5), suggesting that most proteins are resilient to a partial decline in UBA1 function.

Changes in the levels of *Uba1*^RNAi^-modulated proteins may contribute to muscle weakness because of decreased function of downregulated proteins and potential overactivity of upregulated proteins. However, some of the changes in protein abundance induced by *Uba1*^RNAi^ may be harmless or even constitute adaptive (i.e. protective) responses that mitigate the derangement of cellular homeostasis caused by decreased ubiquitination (Rai et al., 2025b). In this respect, it was previously shown that some of the proteins that accumulate in response to knockdown of E2 ubiquitin-conjugating enzymes in HEK293T cells constitute an adaptive response that copes with the cell's decreased ubiquitination capacity (Hunt et al., 2023). Specifically, knockdown of E2 enzymes (and in particular *UBE2D1/2/3*^RNAi^) increases the levels of multiple peroxisomal proteins because their poly-ubiquitination and proteasomal degradation is particularly sensitive to even moderate decline in the activity of E2 ubiquitin-conjugating enzymes (Hunt et al., 2023). These peroxisomal proteins that are upregulated by *E2*^RNAi^ include peroxins (Hunt et al., 2023), which mediate the import of peroxisomal proteins from the cytosol into peroxisomes (Farre et al., 2019). Peroxin upregulation in cells with *E2*^RNAi^ sustains peroxisomal protein import despite the overall decline in ubiquitination, which is otherwise required for peroxisomal protein import by modulating the cyclic activation of Pex5. Therefore, this yin-yang modulation of different components of the import machinery (Pex5 versus other peroxins that form its docking complex) ensures maintenance of peroxisomal protein import in cells with decreased ubiquitination capacity. Analogously, some of the *Uba1*^RNAi^-modulated proteins may contribute to adaptations that preserve muscle homeostasis rather than driving muscle weakness. Interestingly, Pex3 and the peroxisomal enzymes Nudt19/CG10194 and Sgp/FAR are upregulated in response to *Uba1*^RNAi^ in muscle (Figs 2 and 6) and may, therefore, help improve peroxisomal function in this tissue.

Although we have examined the transcriptional changes to identify proteins that are modulated post-transcriptionally (i.e. presumably via ubiquitination) by *Uba1*^RNAi^, it is worth noting that some of the proteins that are modulated independently from mRNA changes include transcription factors, such as Sox8, which was previously found to impair myogenesis (Schmidt et al., 2003). Therefore, some of the transcriptional changes induced by *Uba1*^RNAi^ may arise from *Uba1*^RNAi^-modulated transcription factors and contribute to the muscle wasting and weakness triggered by *Uba1*^RNAi^.

Muscle wasting and weakness are prominent features and the defining cause of death for patients with SMAX2, which is caused by mutations in the *UBA1* gene (Balak et al., 2017; Dlamini et al., 2013; Jędrzejowska et al., 2015; Öztürk et al., 2022; Ramser et al., 2008; Shaughnessy et al., 2020). A recurrent SMAX2-causing mutation is C1731T (Asn577Asn), a silent/synonymous mutation that does not change the UBA1 protein sequence but decreases *UBA1* mRNA levels (Balak et al., 2017; Dlamini et al., 2013; Jędrzejowska et al., 2015; Öztürk et al., 2022; Ramser et al., 2008; Shaughnessy et al., 2020), analogously to what we have experimentally obtained with RNAi-mediated UBA1 knockdown (Figs 1 and 3).

Although the autopsy of a patient with SMAX2 identified potential defects in multiple components of the neuromuscular system (Dlamini et al., 2013), it remains undetermined whether the decline in UBA1 activity in any specific tissue can cause SMAX2-like muscle dysfunction and lethality. By leveraging the genetic tools available in *Drosophila* for muscle-specific interventions, we have found that muscle-restricted knockdown of UBA1 impairs muscle function and survival, indicating that the levels and activity of UBA1 in skeletal muscle are critical.

Although we examined the role of UBA1 in the skeletal muscle of young flies and mice, the ubiquitination cascade ensures muscle homeostasis and protein quality control also during aging (Hunt et al., 2025a, 2021b). Therefore, UBA1 may help maintain an optimal ubiquitination capacity and muscle strength throughout the lifespan. Interestingly, we found that muscle-targeted knockdown of UBA1 shortens lifespan in *Drosophila* (Fig. 1F), suggesting that muscle UBA1 activity may have an organism-wide impact, in agreement with previous studies that identified systemic roles for skeletal muscle (Demontis et al., 2013a; Rai et al., 2021; Rai and Demontis, 2016, 2022; Robles-Murguia et al., 2020). For example, a decline in proteostasis was previously found to reduce lifespan (Jiao et al., 2023; Jiao and Demontis, 2017), whereas muscle-targeted improvements in proteostasis increase longevity (Bai et al., 2013; Demontis et al., 2014; Demontis and Perrimon, 2010).

**Fig. 7.**
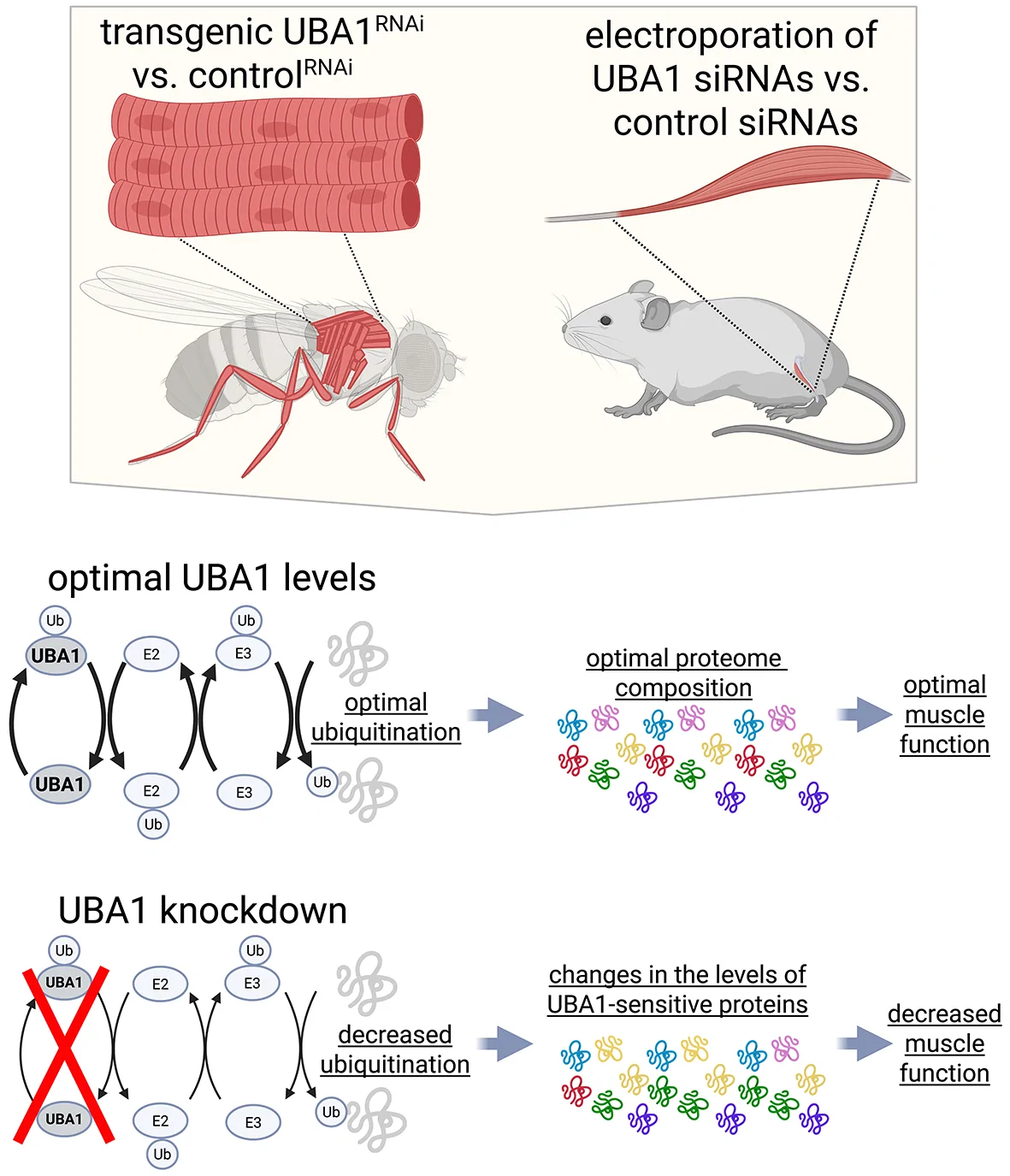
**UBA1 knockdown reduces protein ubiquitination in *Drosophila melanogaster* and mouse skeletal muscles but primarily affects the levels of a limited set of proteins (‘UBA1-sensitive proteins’) that rely on optimal UBA1 levels for their turnover and stability.** Analysis of *Drosophila* skeletal muscles with transgenic knockdown of Uba1 (compared to control *mcherry* RNAi) and mouse TA skeletal muscles electroporated with *Uba1* siRNAs versus control NT siRNAs. In both organisms, a partial knockdown of UBA1 in skeletal muscles reduces protein ubiquitination and elicits transcriptional and proteomic changes. However, despite an overall decline in protein ubiquitination caused by *Uba1* RNAi, analysis of the proteins that are modulated by *Uba1* RNAi without any corresponding changes in mRNA levels indicates that a limited subset of proteins is upregulated and downregulated by UBA1 knockdown: these UBA1-sensitive proteins may constitute ubiquitination targets that require optimal UBA1 levels for their degradation and for their stability, respectively. UBA1 knockdown and changes in the levels of UBA1-sensitive proteins reduce muscle function in *Drosophila* (flight capacity) and mice (muscle mass and force production). In agreement with an important role of skeletal muscle in determining the overall organismal fitness, muscle-restricted *Uba1* RNAi also shortens lifespan in *Drosophila*. The scheme was created in BioRender by Demontis, F. (2026). https://BioRender.com/l0he1ny. This figure was sublicensed under CC-BY 4.0 terms.

In summary, our studies in *Drosophila* and mouse skeletal muscles indicate that UBA1 is a key regulator of muscle function and suggest that reduction of UBA1 function in this tissue is a key driver of SMAX2 progression via the modulation of a limited set of UBA1-sensitive proteins (Fig. 7).

## MATERIALS AND METHODS

### *Drosophila* husbandry and related assays

Flies were maintained at ∼20 flies per tube under controlled conditions of 29°C, 60% humidity and a 12-h light/dark cycle. They were housed in tubes containing cornmeal, soy flour and yeast-based fly food. All experimental assays were conducted using male flies aged 10 days. Lifespan and flight assays were performed following previously established methods (Curley et al., 2024; Hunt et al., 2025a, 2019a, 2021b). The fly stocks used in this study were Mhc-Gal4 (Demontis et al., 2014; Demontis and Perrimon, 2010; Rai et al., 2021; Schuster et al., 1996), UAS-*mcherry*^RNAi^ (#35785), UAS-*Uba1*^RNAi^ (#76066) and UAS-*Uba1*^RNAi#2^ (#25957), obtained from the Bloomington *Drosophila* Stock Center. Human homologs of *Drosophila* proteins were determined with DIOPT.

### qRT-PCR

qRT-PCR was carried out as described in earlier studies (Demontis et al., 2014; Graca et al., 2022, 2021; Hunt et al., 2019b, 2015; Robles-Murguia et al., 2020). Total RNA was isolated using TRIzol reagent (Ambion 15596018), followed by reverse transcription with an iScript cDNA synthesis kit (Bio-Rad). qRT-PCR analysis was performed using SYBR Green dye (Bio-Rad) on a Bio-Rad CFX96 instrument. The comparative CT method was applied for relative mRNA quantification, normalizing against *Hprt* levels in mice (Hunt et al., 2015) and *Tub84B* levels in *Drosophila* (Hunt and Demontis, 2022). The following oligonucleotides were used for qRT-PCR: *Uba1* (mouse), 5′-AGGTGGTTTGCTCTTCCGTT-3′ and 5′-CGTTCTTCGCCATTCCGTTG-3′; *Hprt* (housekeeping mouse control), 5′-GATTAGCGATGATGAACCAGGTT-3′ and 5′-TCCAAATCCTCGGCATAATGAT-3′; *Uba1* (*Drosophila*), 5′-ACAGAAGGTTAAGGTGCCCG-3′ and 5′-TGGCGTGATCTTGGAGCTTT-3′; *Tub84B* (housekeeping *Drosophila* control), 5′-GTTTGTCAAGCCTCATAGCCG-3′ and 5′-GGAAGTGTTTCACACGCGAC-3′.

### RNA-seq

RNA-seq was performed as previously described (Curley et al., 2024; Graca et al., 2022, 2023b; Robles-Murguia et al., 2019). *Drosophila* thoraces (primarily composed of skeletal muscle) and mouse TA muscles were used for these preparations. In brief, RNA-seq samples were prepared using TRIzol (Ambion 15596018), followed by RNA extraction through isopropanol precipitation from the aqueous phase. Libraries were constructed from 1 μg total RNA using the Illumina TruSeq RNA Sample Prep v2 Kit, following the manufacturer's instructions. Sequencing was carried out on the Illumina NovaSeq 6000 platform, generating 100-bp paired-end reads. These reads were quality trimmed and filtered to retain sequences with a Phred score of 20 or higher and lengths of at least 50 bp. The processed reads were then aligned to the *D. melanogaster* reference genome (BDGP6/dm6) and the mouse reference genome (GRCm39/mm39) using CLC Genomics Workbench version 20.0.4 (Qiagen). Gene expression levels were quantified as transcripts per million (TPM) using the CLC RNA-Seq Analysis tool. Differential expression was evaluated through non-parametric ANOVA, employing Kruskal–Wallis and Dunn's multiple comparisons tests on log-transformed TPM data within Partek Genomics Suite version 7.0 (Partek Inc.). The RNA-seq datasets generated in this study are available in the NCBI Gene Expression Omnibus (GEO) repository under accession number GSE305834. GO enrichment analysis was conducted using DAVID.

### Protein sample preparation, digestion and TMT labeling

*Drosophila* thoraces (primarily composed of skeletal muscle) and mouse TA muscles were homogenized using a bullet blender with 0.5-mm zirconium oxide beads (Next Advance ZROB05) in RIPA buffer (Cell Signaling Technology 9806) supplemented with 8 M urea. The homogenates were centrifuged (13,000 ***g***), and the resulting supernatants were used for downstream analyses. For TMT labeling (Bai et al., 2017, 2021, 2020; Dey et al., 2019; Jiao et al., 2021; Nuga et al., 2024; Wang et al., 2020; Xu et al., 2009b; Yu et al., 2021), tissue lysates were resolved by SDS-PAGE, and protein concentrations were determined using Coomassie Blue-stained short gels with bovine serum albumin (BSA; Sigma-Aldrich 9048-46-8) as the standard (Xu et al., 2009a). Gel bands were excised, washed twice in 50% acetonitrile (Honeywell Research Chemicals AH015-4) and dried. Proteins were digested overnight with trypsin (Promega V5113) at a 1:10 enzyme-to-substrate ratio in 5 mM HEPES (pH 8.5; Sigma-Aldrich H3375). Peptides were extracted, dried, resuspended in 50 mM HEPES (pH 8.5) and labeled using TMTpro 18-plex reagents (Thermo Fisher Scientific A52045) as per the manufacturer's instructions.

### Two-dimensional high-performance liquid chromatography and MS

TMT-labeled peptides were pooled in equal amounts, desalted and fractionated via offline high-performance liquid chromatography (HPLC; Agilent 1220) using basic pH reverse-phase chromatography (Xbridge C18 column, 4.6 mm×25 cm, 3.5 μm; Waters 186003943). Fractions were dried and reconstituted in 5% formic acid for analysis by liquid chromatography (LC)-tandem mass spectrometry (MS/MS) at acidic pH. Samples were separated on a nanoscale reverse-phase C18 column (Easy-Spray PepMap RSLC, 2 μm, 15 cm×75 μm; Thermo Fisher Scientific ES904) using an Ultimate 3000 HPLC system and eluted over a 90- to 120-min gradient. Peptides were ionized via electrospray and analyzed on an Orbitrap Exploris 480 mass spectrometer in data-dependent mode. Survey scans were acquired in the Orbitrap [60,000 resolution, 1×10^6^ automatic gain control (AGC) target, 50 ms max ion time], followed by high-resolution MS/MS scans (60,000 resolution, 2×10^5^ AGC target, 120 ms max ion time, 32 higher-energy collisional dissociation, 1 m/z isolation window, 15 s dynamic exclusion).

### MS data analysis

MS/MS raw files were analyzed using the JUMP hybrid tag-based search engine (Wang et al., 2014). Spectra were searched against UniProt *Drosophila* and mouse protein databases, concatenated with a reverse decoy database to estimate false discovery rates (FDRs). Search parameters included 15-ppm mass tolerance for precursor and product ions, fully tryptic peptides (allowing two missed cleavages), up to three variable modification sites, and consideration of a, b and y ions. Static modifications included TMT tags on lysines and peptide N-termini (+304.20715 Da); methionine oxidation (+15.99492 Da) was treated as a dynamic modification. MS/MS matches were filtered by mass accuracy and scoring to achieve ∼1% protein FDR. Protein quantification was performed by summing TMT reporter ion intensities across all matched peptide-spectrum matches using the JUMP software suite (Wang et al., 2014), which was used for the statistical analysis of the data with limma (moderated *t*-statistics). The MS data were deposited in the ProteomeXchange Consortium via PRIDE with the dataset identifier PXD067470.

### Western blotting

Similar procedures as described before (Hunt et al., 2025b, 2015; Rai et al., 2025a) were utilized. Protein extracts were prepared from *Drosophila* thoraces and mouse TA muscles by homogenization in 200 μl in RIPA buffer (Cell Signaling Technology 9806) supplemented with 8 M urea. Samples were mixed with SDS-Blue loading buffer (Cell Signaling Technology 7722) and 0.1 M DTT (Cell Signaling Technology 1425S), then boiled at 95°C for 5 min. Proteins were separated on 4-20% SDS-PAGE gels (Bio-Rad 4561096), alongside molecular mass markers (Bio-Rad 1610374) and transferred onto PVDF membranes (Millipore IPVH00010), which were stained with Ponceau S. Membranes were blocked with 5% milk or BSA for 1 h and incubated overnight at 4°C with primary antibodies (1:1000 dilution) against ubiquitin (Santa Cruz Biotechnology, P4D1 sc-8017), UBA1 (UBE1a/b; Cell Signaling Technology 4891), Gyg1 (Santa Cruz Biotechnology, E-11 sc-271109) and α-tubulin (Cell Signaling Technology 2125). Detection was performed using appropriate secondary antibodies. The full scans of western blots are provided within Dataset 6.

### Electroporation of siRNAs into mouse TA muscles

Male C57BL/6J mice (JAX#000664, The Jackson Laboratory) were housed in the Animal Resource Center at St. Jude Children's Research Hospital and maintained on a standard chow diet. All procedures were approved by the St. Jude Institutional Animal Care and Use Committee (IACUC). Electroporation of siRNAs into the TA muscles of 3-month-old male mice was performed as previously described (Donà et al., 2003; Hunt et al., 2021b; Sandri et al., 2003, 2004; Stephan et al., 2022, 2026a) by utilizing a mixture of four distinct ON-TARGETplus siRNA species (SMARTpool) for the same target, a strategy that provides higher knockdown potency and specificity while minimizing off-target effects (Anderson et al., 2008). One TA muscle was injected with a siRNA SMARTpool targeting UBA1 (Horizon Discovery L-062420-01) while the contralateral TA from the same mouse was electroporated with a control non-targeting (NT) siRNA SMARTpool (Horizon Discovery D-001810-10). Mice were anesthetized with isoflurane, and hair was removed from the hindlimbs. TA muscles were pretreated with 30 μl of 0.4 U/μl hyaluronidase (Sigma-Aldrich 4272) using a 29G1/2 insulin syringe. After a 2-h recovery period, the mice were re-anesthetized, and 1500 pmol siRNAs in 30 μl Dharmacon's recommended resuspension buffer (60 mM KCl, 6 mM HEPES, pH 7.5 and 0.2 mM MgCl_2_) were injected into the TA muscle. One leg received NT siRNAs, while the contralateral leg was injected with *Uba1* siRNAs. Electroporation was performed using an ECM830 Electro Square Porator (BTX Harvard Apparatus) and 10-mm gold tip electrodes (BTX Genetrodes 45-0114), first with four pulses at 80 V/cm for 20 ms at 1 Hz, followed by four additional pulses after reorienting the electrodes perpendicularly to the tibia. Mice were sacrificed 7 days post-electroporation, and TA muscles were collected and snap frozen for further analysis. RNA-seq and TMT MS data were all obtained from muscles collected 1 week after siRNA electroporation.

### Skeletal muscle force measurement in mice

The twitch and tetanic force produced by the TA muscle were measured as described previously (Graca et al., 2023a; Hunt et al., 2021b, 2019b; Liu et al., 2016). Mice were deeply anesthetized with isoflurane, and vital signs were continuously monitored. The distal TA tendon was carefully isolated and tied with braided surgical silk (5/0, 19 mm, reverse cutting). All the branches of the sciatic nerve were transected except for the common peroneal branch. The foot was secured to a platform, and the knee was immobilized using a stainless-steel pin. Body temperature was maintained at 37°C. The tendon was attached to a 305B dual-mode servomotor transducer (Aurora Scientific), and muscle contractions were induced by stimulating the sciatic nerve using bipolar electrodes and supramaximal square-wave pulses (0.2 ms; 701A stimulator, Aurora Scientific). Data acquisition and servomotor control were performed with the DMC software (version 5.202, Aurora Scientific). Optimal muscle length was determined by gradually stretching the muscle to achieve maximal isometric twitch force.

### Immunostaining and confocal microscopy of mouse skeletal muscles

Analysis of myofiber size, type and number was conducted following established protocols (Graca et al., 2022, 2023a,b; Hunt et al., 2021b, 2019b; Stephan et al., 2026b). Unfixed cryosections of TA muscle were incubated for 1 h in a blocking solution of PBS containing 2% BSA and 0.1% Triton X-100, followed by overnight incubation at 4°C with primary antibodies diluted 1:150. The following primary antibodies were used: mouse IgG1 anti-myosin heavy chain type 2a [Developmental Studies Hybridoma Bank (DSHB) SC-71], mouse IgM anti-myosin heavy chain type 2b (DSHB BF-F3) and rat anti-laminin α2 (Santa Cruz Biotechnology, 4H8-2 sc-59854). Following washes, sections were incubated with secondary antibodies (1:200): anti-mouse IgG1 Alexa Fluor 488 (Life Technologies A21121), anti-mouse IgM Alexa Fluor 555 (Life Technologies A21426), anti-rat IgG Alexa Fluor 647 (Life Technologies A21247). Imaging was performed using a Nikon confocal microscope, and 10× images were stitched to compile a full muscle cross-section. Myofiber boundaries were detected by inverse thresholding of laminin α2 staining, and fiber types were identified based on MHC signals: type 2a (green), type 2b (red) and presumptive type 2x (unstained/black). Myofiber size was automatically measured by Feret's minimal diameter, which provides a robust metric for irregular or non-circular fiber shapes (Bloemberg and Quadrilatero, 2012). Total myofiber counts were obtained by automatic segmentation of laminin α2-stained borders across entire cross-sections with the Nikon Elements software.

### Statistical analysis

Data analysis, plotting and statistical testing were conducted using Microsoft Excel (v14.7.3), GraphPad Prism (v10) and SRplot. Unpaired two-tailed Student's *t*-tests were used for the comparisons between two groups. Paired two-tailed Student's *t*-tests were used when comparing matched contralateral TA muscles. One-way ANOVA followed by post-hoc testing was applied for the statistical analysis of more than two groups. The lifespan data were analyzed with log-rank tests. The number of biological replicates (*N*) is indicated in the figure legends and represents independently generated muscle samples pooled from multiple flies or individual TA muscles sourced from distinct mice. Bar graphs are presented as mean±s.d. or ±s.e.m., as indicated. *P*<0.05 was considered statistically significant.

## Supplementary Material

10.1242/dmm.052935_sup1 Supplementary information

Dataset 1. RNA-seq of Drosophila skeletal muscle with muscle-specific Uba1 knockdown vs. control mcherry knockdown.

Dataset 2. TMT mass spec analyses of Drosophila skeletal muscle with Uba1 knockdown vs. control mcherry knockdown.

Dataset 3. Significant transcriptional changes induced by UBA1 knockdown in Drosophila and mouse skeletal muscles.

Dataset 4. RNA-seq of mouse skeletal muscle electroporated with UBA1 siRNAs vs. control NT (non-targeting) siRNAs.

Dataset 5. TMT mass spec analyses of mouse skeletal muscle electroporated with UBA1 siRNAs vs. control NT (non-targeting) siRNAs.

Dataset 6. Source data file and full scans of western blots.
