# Supplementary material for: UBA1 knockdown dysregulates UBA1-sensitive proteins and impairs muscle function in models of spinal muscular atrophy X-linked 2
Source: Dis Model Mech. 2026 Aug 26;19(8):dmm052935. doi: 10.1242/dmm.052935 (PMC13580589; doi:10.1242/dmm.052935)
Supplement: Supplementary file 1 [file dmm-19-052935-s1.pdf]

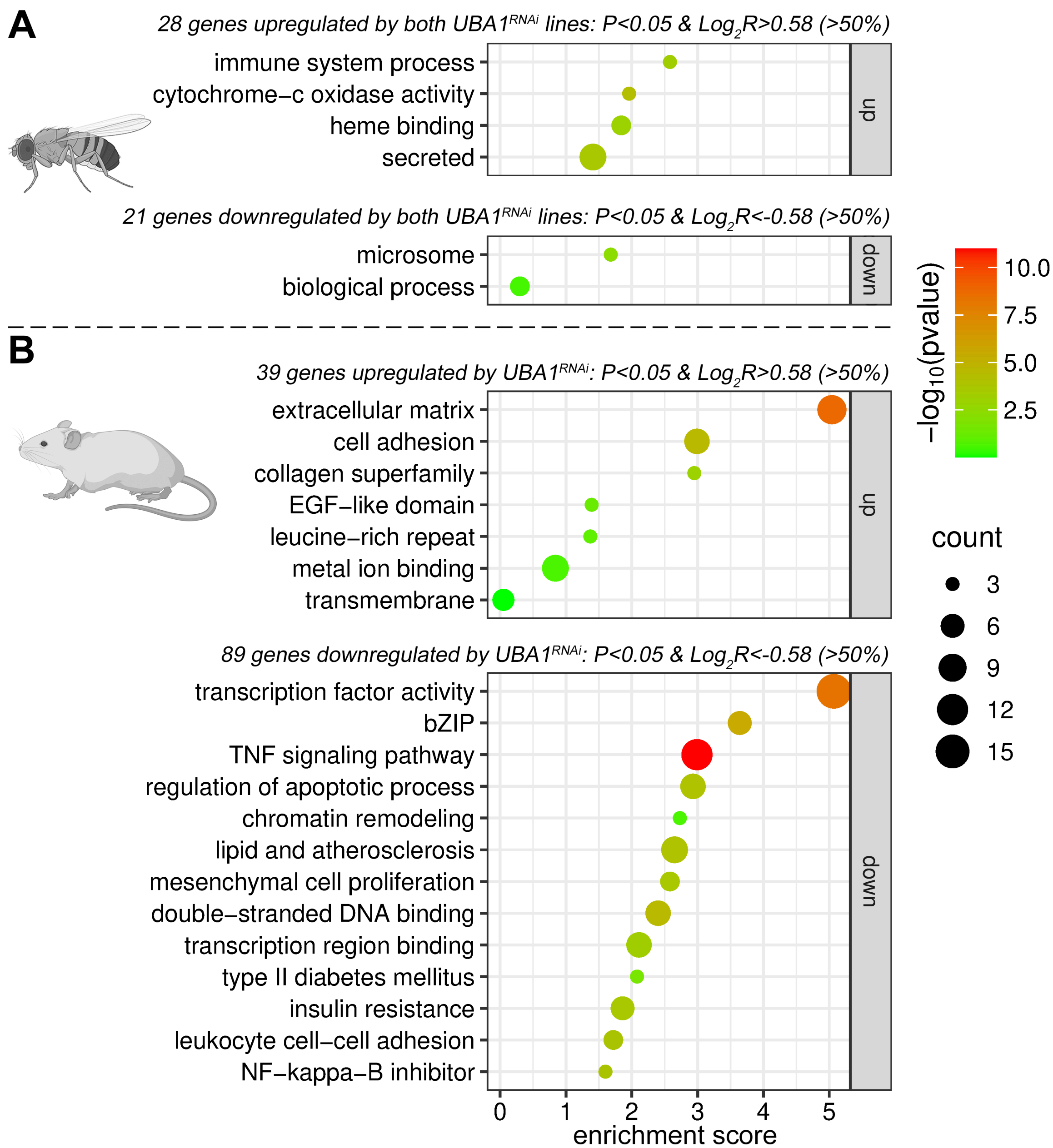


**Fig. S1. Gene expression changes induced by UBA1 knockdown in Drosophila and mouse skeletal muscle.** GO term categories that describe the genes that are transcriptionally upregulated and downregulated by UBA1 knockdown in Drosophila (A) and mouse (B) skeletal muscles. The gene counts, enrichment scores, and -$\log_{10}$(*P*-value) are indicated. There are respectively 28 and 21 genes that are consistently up- and down-regulated in Drosophila by both UBA1 RNAi lines with a significance of $P<0.05$ and a 50% cut-off, i.e. $\text{Log}_2\text{R}>|0.58|$. In mouse skeletal muscle, there are 39 and 89 genes that are significantly ($P<0.05$) up- and down-regulated with $\text{Log}_2\text{R}>|0.58|$ by UBA1 siRNAs vs. control NT (non-targeting) siRNAs. Note that no gene is commonly upregulated or downregulated by UBA1 knockdown in Drosophila and mouse skeletal muscle. See also Dataset S3. Fly and mouse schemes were created in BioRender by Demontis, F. (2026). https://BioRender.com/1i8vuo7. This figure was sublicensed under CC-BY 4.0 terms.

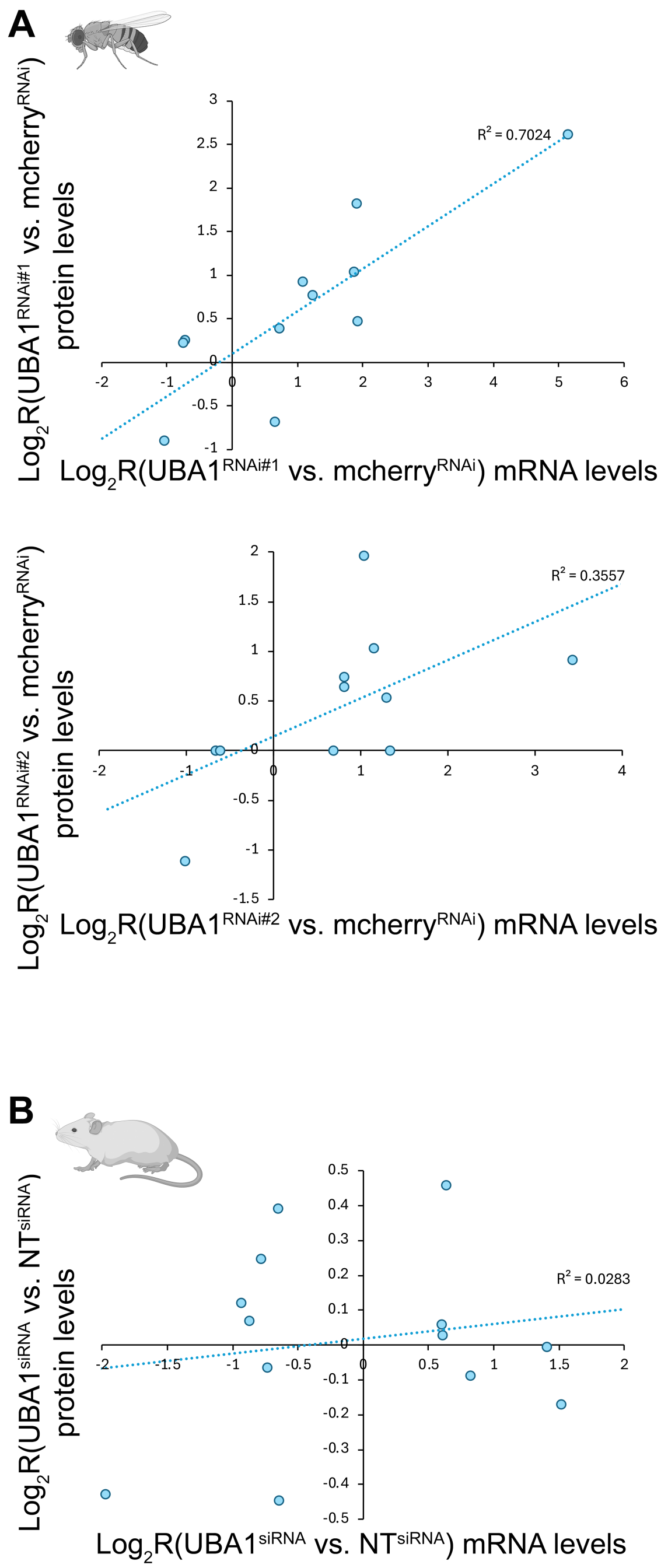


**Fig. S2. Protein levels corresponding to significantly regulated mRNAs in Drosophila and mouse skeletal muscles with UBA1 knockdown.** Comparison of the mRNAs that are significantly modulated by UBA1 knockdown in Drosophila (A) and mouse (B) skeletal muscles and the corresponding protein levels detected by TMT mass spectrometry. There are relatively high $R^2$ values ($R^2$=0.7024 and $R^2$=0.3557) for Drosophila UBA1$^{RNAi\#1}$ and UBA1$^{RNAi\#2}$, indicating that many of the significantly modulated genes lead to corresponding protein level changes. However, $R^2$ values are low in mouse skeletal muscle ($R^2$=0.0283), indicating that mRNAs that are significantly regulated overall do not lead to corresponding protein changes, presumably because of compensatory modulation by mRNA translation or protein degradation. Dataset S6. Fly and mouse schemes were created in BioRender by Demontis, F. (2026). https://BioRender.com/1i8vuo7. This figure was sublicensed under CC-BY 4.0 terms.

**Dataset 1. RNA-seq of Drosophila skeletal muscle with muscle-specific Uba1 knockdown vs. control mcherry knockdown.**

Available for download at
https://journals.biologists.com/dmm/article-lookup/doi/10.1242/dmm.052935#supplementary-data

**Dataset 2. TMT mass spec analyses of Drosophila skeletal muscle with Uba1 knockdown vs. control mcherry knockdown.**

Available for download at
https://journals.biologists.com/dmm/article-lookup/doi/10.1242/dmm.052935#supplementary-data

**Dataset 3. Significant transcriptional changes induced by UBA1 knockdown in Drosophila and mouse skeletal muscles.**

Available for download at
https://journals.biologists.com/dmm/article-lookup/doi/10.1242/dmm.052935#supplementary-data

**Dataset 4. RNA-seq of mouse skeletal muscle electroporated with UBA1 siRNAs vs. control NT (non-targeting) siRNAs.**

Available for download at
https://journals.biologists.com/dmm/article-lookup/doi/10.1242/dmm.052935#supplementary-data

**Dataset 5. TMT mass spec analyses of mouse skeletal muscle electroporated with UBA1 siRNAs vs. control NT (non-targeting) siRNAs.**

Available for download at
https://journals.biologists.com/dmm/article-lookup/doi/10.1242/dmm.052935#supplementary-data

**Dataset 6. Source data file and full scans of western blots.**

Available for download at

https://journals.biologists.com/dmm/article-lookup/doi/10.1242/dmm.052935#supplementary-data
